# Treatment of Aphthæ

**Published:** 1847-05

**Authors:** 


*Treatment of Aphthæ.*—Professor Lippich, of Padua, uses with advantage the following application in the treatment of aphthæ: Honey, 15 parts; sulphuric acid, 1 part (by weight). The mixture is applied by means of a camel's hair brush to the aphthous ulcerations.—*Gaz. Méd.*—*Ranking's Abstract.*

